# Synthesis, crystal structure and Hirshfeld surface analysis of *N*-(4-meth­oxy­phen­yl)picolinamide

**DOI:** 10.1107/S2056989024010843

**Published:** 2024-11-14

**Authors:** Dilnoza Burieva, Batirbay Torambetov, Sarvinoz Bobonazarova, Anvar Abdushukurov, Tursinali Kholikov, Akram A Khan, Jamshid Ashurov, Mukhriddin Yusufov

**Affiliations:** a National University of Uzbekistan named after Mirzo Ulugbek 4 University St Tashkent 100174 Uzbekistan; b Physical and Material Chemistry Division CSIR-National Chemical Laboratory,Pune 411008 India; c Institute of Bioorganic Chemistry, Academy of Sciences of Uzbekistan, M. Ulugbek, St, 83, Tashkent, 100125, Uzbekistan; Indian Institute of Science Education and Research Bhopal, India

**Keywords:** crystal structure, mol­ecular structure, picolinic acid, picolinamide, Hirshfeld surface analysis

## Abstract

The synthesis, crystal structure, and Hirshfeld surface analysis of *N*-(4-meth­oxy­phen­yl)picolinamide (MPPA), C_13_H_12_N_2_O_2_, are presented. MPPA crystallizes in the monoclinic space group *P*2_1_/*n*, with a single mol­ecule in the asymmetric unit. Structural analysis reveals that all non-hydrogen atoms are nearly coplanar, and the mol­ecule exhibits two intra­molecular hydrogen bonds that stabilize its conformation. Supra­molecular features include significant inter­molecular inter­actions, primarily C—H⋯π and various hydrogen bonds, contributing to the overall crystal cohesion, as confirmed by energy framework calculations yielding a total inter­action energy of −138.3 kJ mol^−1^. Hirshfeld surface analysis indicates that H⋯H inter­actions dominate, followed by C⋯H and O⋯H inter­actions, highlighting the role of van der Waals forces and hydrogen bonding in crystal packing.

## Abstract 2

The mol­ecular and crystal structure of *N*-(4-meth­oxy­phen­yl)picolinamide were studied and Hirshfeld surfaces and fingerprint plots were generated to investigate various inter­molecular inter­actions.

## 1. Chemical context

The synthesis of amide derivatives of carbonic acid is a vital area of organic chemistry, owing to the widespread presence and significance of amide bonds in various applications. These bonds are fundamental components in polymers such as nylon, proteins, and peptides, as well as in natural products such as paclitaxel and penicillin (Valeur & Bradley, 2009 ▸). Notably, approximately 25% of pharmaceuticals contain at least one amide bond, highlighting their importance in drug development (Ghose *et al.*, 1999 ▸; Kamanna *et al.*, 2020 ▸; Goodreid *et al.*, 2014 ▸).

Amide-linked compounds are typically synthesized through acyl­ation methods, often involving acyl chlorides or coupling agents that facilitate the reaction between carb­oxy­lic acids and amines (Montalbetti & Falque, 2005 ▸; Pon *et al.*, 1999 ▸; Han & Kim, 2004 ▸; Valeur & Bradley, 2009 ▸). Moreover, the use of orthoboric acid and organoboronic compounds has gained prominence for direct amide synthesis, demonstrating their effectiveness as catalysts in these reactions (Tang, 2005 ▸).

Among the diverse array of organic substances, heterocyclic compounds are particularly significant, especially heterocyclic aromatic compounds, which play crucial roles in biological systems. Pyridine and its derivatives are key representatives of this class (Kaiser *et al.*, 1996 ▸). Specifically, 2-pyridine­carb­oxy­lic acid amides are noteworthy for their versatility as reagents and catalysts in various organic syntheses. Their strong ligand properties in coordination chemistry have also been extensively studied (Sambiagio *et al.*, 2016 ▸).

The combination of a pyridine fragment with an amide bond not only enhances the reactivity of these compounds but also expands their applicability across multiple sectors of the chemical industry. The nitro­gen atom in the pyridine ring possesses an unshared electron pair, which, along with the electron-rich carbonyl group of the amide, facilitates the formation of coordination bonds with various metals (Mishra *et al.*, 2008 ▸; Almodares *et al.*, 2014 ▸; Wang *et al.*, 2019 ▸; Basri *et al.*, 2017 ▸). This inter­action paves the way for the development of complex compounds with tailored properties, making these derivatives integral to advancements in both synthetic and applied chemistry.
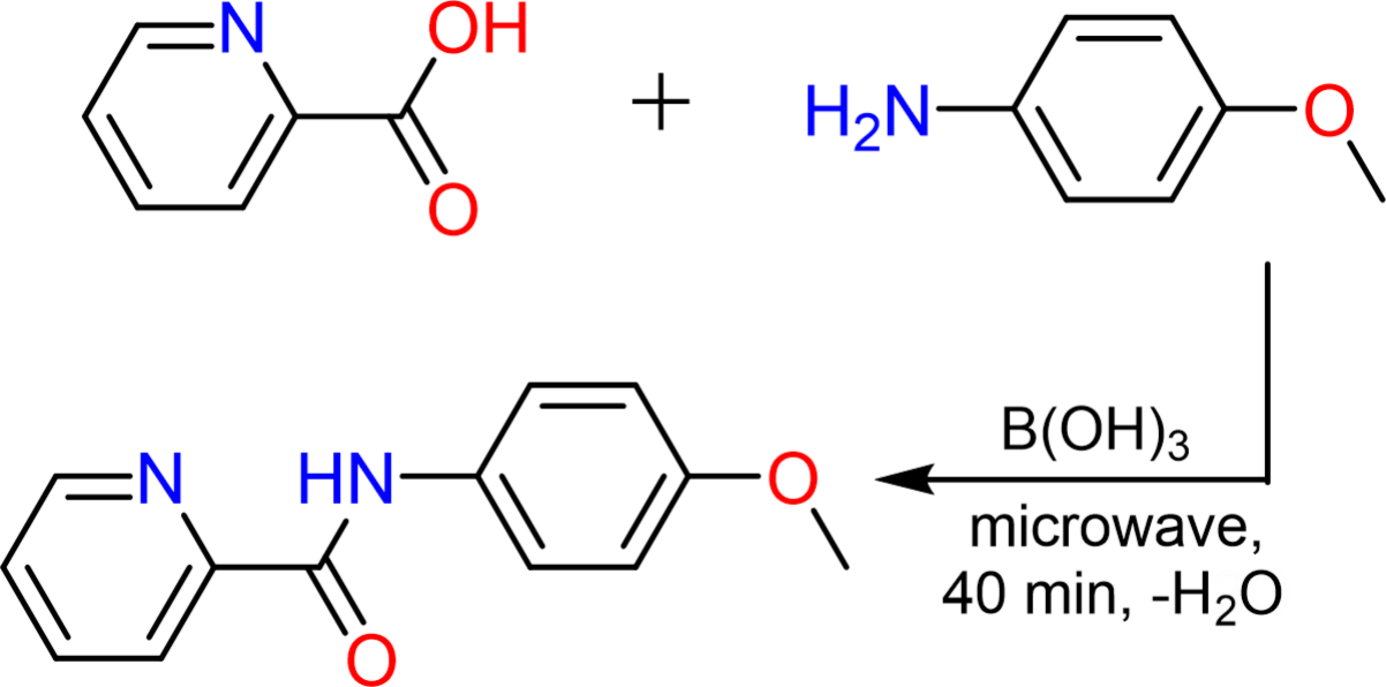


## 2. Structural commentary

**Figure 1:**
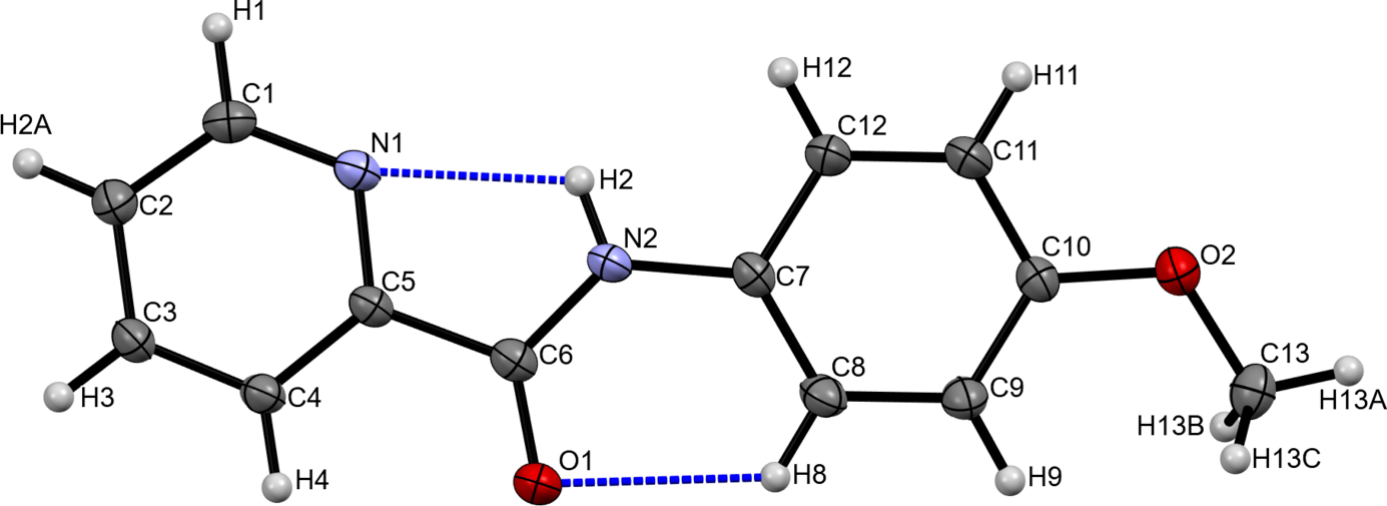
A view of the mol­ecular structure of MPPA, showing the atom labelling. Displacement ellipsoids are drawn at the 30% probability level. Intra­molecular hydrogen bonds are shown as dashed lines.

*N*-(4-Meth­oxy­phen­yl)picolinamide (MPPA) crystallizes in the primitive centrosymmetric monoclinic space group *P*2_1_/*n*. The asymmetric unit consists of a single mol­ecule of MPPA (Fig. 1 ▸). All atoms, except for the hydrogen atoms, lie nearly in a plane, with a maximum deviation of 0.195 Å for atom C11. The dihedral angle between the mean planes of the pyridine ring (C1–C5/N1) and the benzene ring (C7–C12) is 14.25 (5)°. The torsion angles for the groups N1—C5—C6—N2 and C6—N2—C7—C8 are 3.1 (4)° and 12.7 (6)°, respectively. The distance between C6 and O1 in the amide moiety is 1.233 (4) Å (Shen *et al.*, 2019 ▸; Razzoqova *et al.*, 2022 ▸). The conformation of the meth­oxy group is nearly planar with respect to the benzene ring, with a C9—C10—O2—C13 torsion angle of 7.5 (5)°. The nitro­gen atom (N2) in the imino group is also planar, with the sum of the bond angles around it being equal to 360°. There are two intra­molecular hydrogen bonds: one between the pyridine nitro­gen (N1) and the amide nitro­gen (N2) (N2—H2⋯N1 = 2.18 Å), and another between the amide oxygen (O1) and the benzene carbon (C8) (C8—H8⋯O1 = 2.37 Å). These inter­actions form *S*(5) and *S*(6) ring motifs, respectively (Bernstein *et al.*, 1995 ▸), which contribute to the stabilization of the mol­ecular conformation (Fig. 1 ▸, Table 1 ▸).

## 3. Supra­molecular features and energy framework calculations

**Figure 2:**
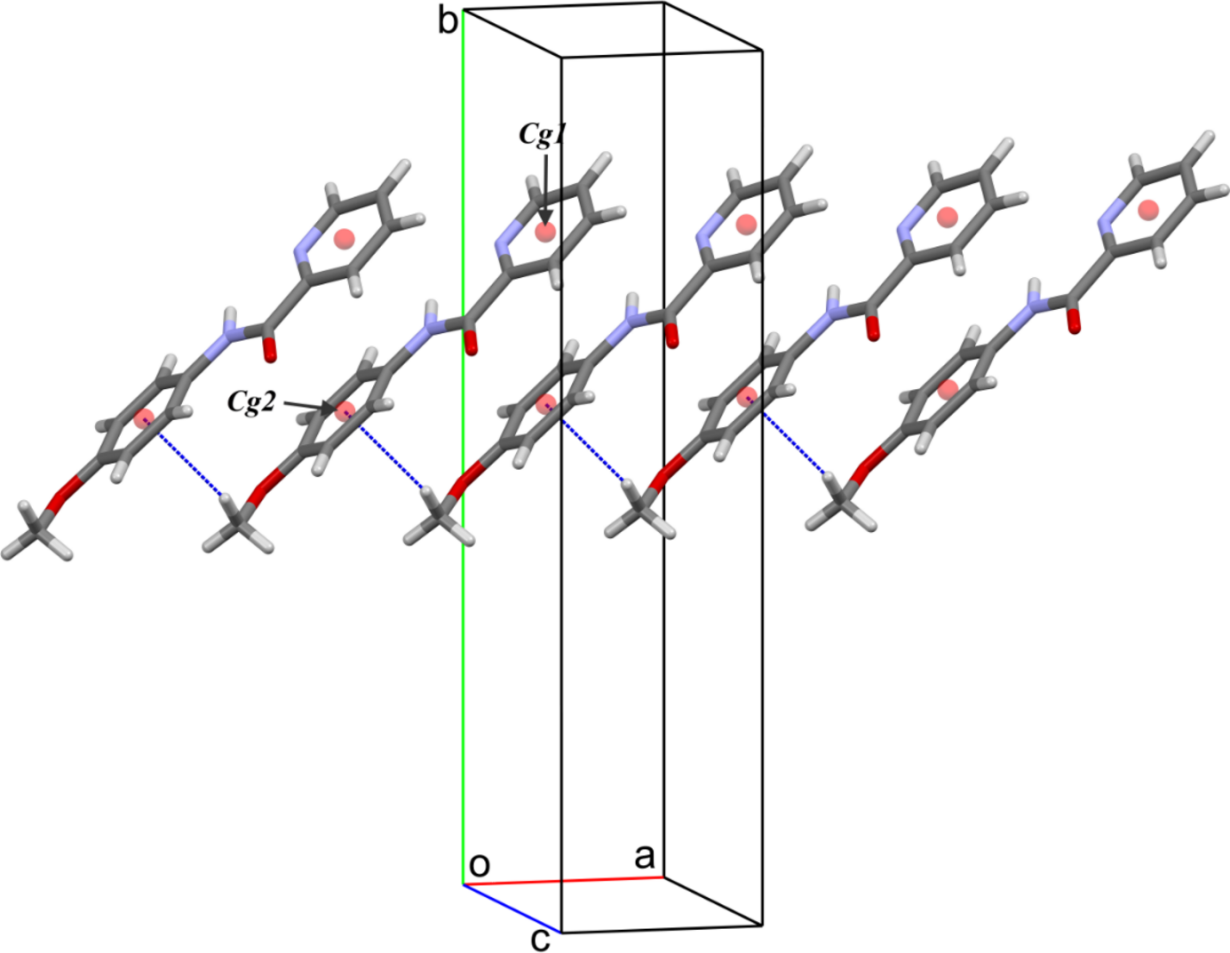
Crystal packing of MPPA along the [100] direction. C—H⋯*Cg* inter­actions are shown as cyan dashed lines.

**Figure 3:**
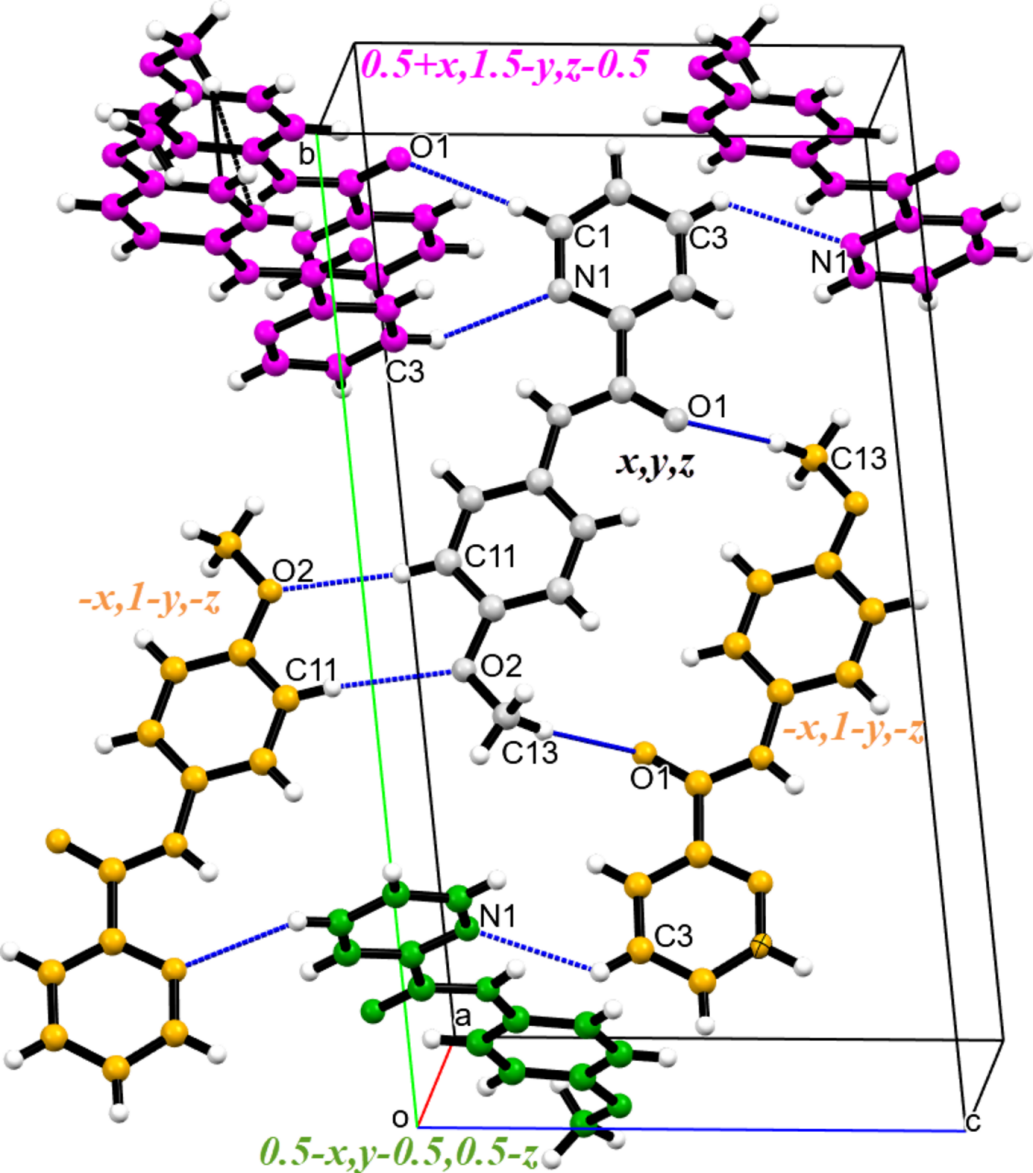
Crystal packing of MPPA in a projection along the [100] direction. Hydrogen bonds are shown as cyan lines. The colour codes of the atoms participating in inter­actions and corresponding symmetry operations of neighbours are similar.

In the crystal structure, mol­ecules are inter­connected through weak inter­actions. Notably, an inter­molecular C—H⋯π inter­action (C13—H13*B*⋯*Cg*1) involves the centroid of the C7–C12 benzene ring (see Table 1 ▸, Fig. 2 ▸). These inter­actions are crucial for the packing of mol­ecules along the *a*-axis direction. Additionally, several weak hydrogen bonds further contribute to the structural integrity. These include C1—H1⋯O1, C3—H3⋯N1, C11—H11⋯O2 and C13—H13⋯O1 and help consolidate the mol­ecules along the *b*- and *c*-axis directions (see Table 1 ▸, Fig. 3 ▸). Notably, the C11—H11⋯O2 inter­action results in the formation of inversion dimers, creating closed eight-membered rings characterized by an 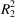
(8) graph-set motif (Etter, 1990 ▸).

**Figure 4:**
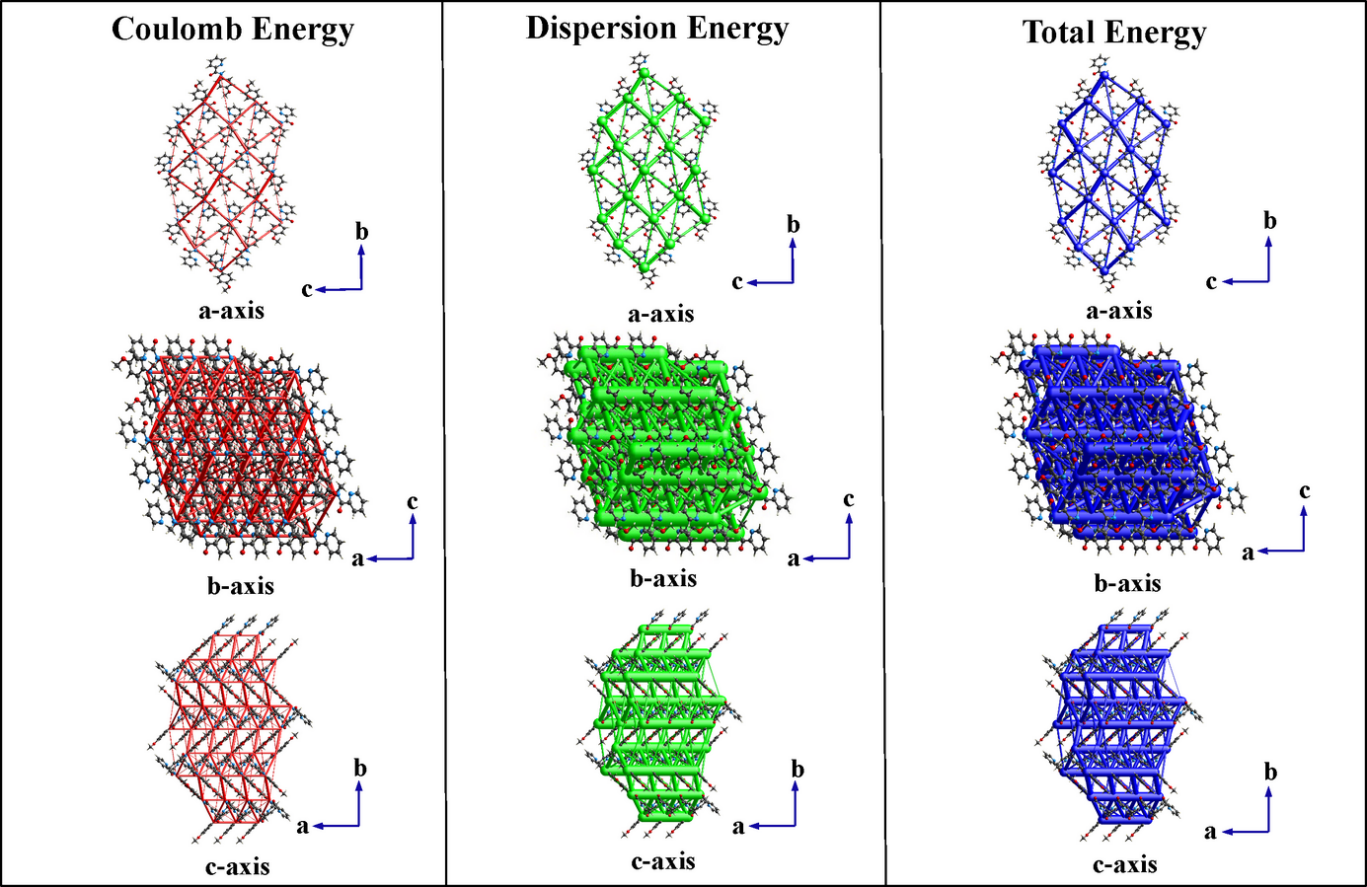
Energy framework calculations of the MPPA crystal are observed along the *a*, *b* and *c* axes.

To identify the inter­molecular inter­actions of MPPA, energy framework calculations were carried out using *CrystalExplorer* (Spackman *et al.*, 2021 ▸). The wavefunctions were derived from the SCXRD CIF file using the Gaussian B3LYP-D2/6-31G(d,p) method. The total inter­action energy (*E*_tot_) was calculated by combining the electrostatic (*E*_ele_), polarization (*E*_pol_), dispersion (*E*_dis_) and repulsion (*E*_rep_) contributions, giving a value of −138.3 kJ mol^−1^. Electrostatic and dispersion forces are the dominant contributors to the stability of the crystal, particularly along the *a*-axis direction (Fig. 4 ▸). Inter­action energies were computed for mol­ecules within a 3.8 Å radius of a reference mol­ecule, omitting those below 5 kJ mol^−1^ for clarity. In the energy framework visualization, thick cylinders represented stronger inter­actions, allowing easy identification of significant inter­molecular inter­actions (Fig. 4 ▸).

## 4. Hirshfeld surface analysis

**Figure 5:**
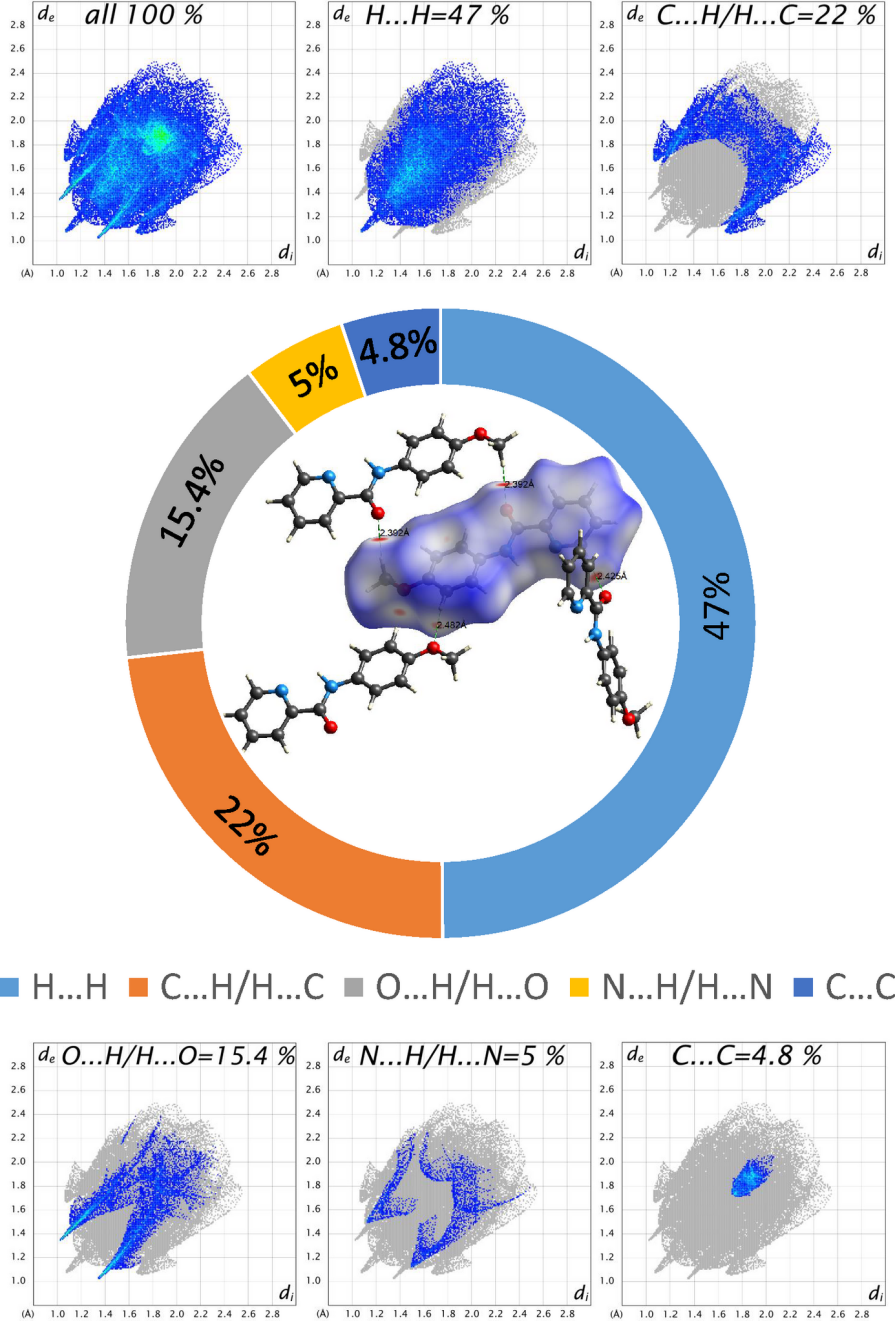
View of the three-dimensional Hirshfeld surfaces plotted over *d*_norm_ and contributions of the various contacts to the two-dimensional fingerprint plots of the MPPA mol­ecule.

The studies carried out on the Hirshfeld surface show that H⋯H inter­actions are the most abundant at 47% of the total inter­mol­ecular inter­actions, suggesting the importance of van der Waals inter­actions in the structural organization of the crystal due to the hydrogen atoms. The C⋯H inter­actions come second, accounting for 22% and signify the presence of weak dispersive forces or possible C—H⋯π inter­actions, which further help stabilize the crystal. The 15.4% contribution of O⋯H inter­actions indicate the presence of strong hydrogen-bonding inter­actions between oxygen atoms and hydrogen atoms, which play a key role in the crystal packing. The smallest contributions to the crystal packing are from N⋯H (5%), C⋯C (4.8%), C⋯N (3.4%) and C⋯O (1.9%) contacts (Fig. 5 ▸).

## 5. Database survey

A search of the Cambridge Structural Database (CSD, Version 5.45, last updated March 2024; Groom *et al.*, 2016 ▸) did not find any structures for the synthesized MPPA. However, two complex compounds were identified in which MPPA acts as a bidentate ligand, coordinating with Co and Rh metals (BAKQIR, Ghandhi *et al.*, 2021 ▸; BEDSAG, Bhattacharya *et al.*, 2012 ▸). The authors also reported structures of various picolinamides, including several benzene derivatives. Among these, mono-substituted derivatives such as *o*-, *m*-, and *p*-mono­chloro (KEHWED, KEHWAZ, GEPQIC; Gallagher *et al.*, 2022 ▸;), *p*-fluoro (KAHRUI; Wilson & Munro 2010 ▸), p-bromo (WUVYIV; Qi *et al.*, 2003 ▸) , *p*-hy­droxy (LUGPOV; Ali *et al.*, 2014 ▸), *p*-nitro (KAHSAP; Wilson *et al.*, 2010 ▸), and *o*-, *m*-, and *p*-methyl (UXEYOM, UXEYIG, UXEYEC; Mocilac & Gallagher, 2011 ▸) picolinamides were documented. In these mol­ecules, the mean planes of the pyridine and benzene rings are generally nearly coplanar, with slight twisting in some cases. Notably, the *p*-fluoro and *p*-methyl derivatives exhibit significant twisting, with dihedral angles of 36.26 and 33.63°, respectively.

## 6. Synthesis and crystallization

A mixture of 0.615 g (0.005 mol) of picolinic acid, 1.23 g (0.01 mol) of *p*-anisidine, and 0.31 g (0.005 mol) of orthoboric acid was thoroughly combined and placed in a reaction flask. The flask was then subjected to microwave irradiation for 40 minutes. Upon completion of the reaction, a 10% NaHCO_3_ solution was added to the mixture, and the resulting solid was filtered off. The filtrate was recrystallized using a 30% ethanol–water solution, yielding 0.798 g (70%) of the final product, m.p. 362–363 K.

^1^H NMR (600 MHz, CDCl_3_) (J, Hz): δ 9.92 (*s*, 1H), 8.60 (*ddt*, *J* = 4.8, 1.7, 0.8 Hz, 1H), 8.29 (*dt*, *J* = 7.8, 1.1 Hz, 1H), 7.92–7.86 (*m*, 1H), 7.73–7.67 (*m*, 2H), 7.49–7.44 (*m*, 1H), 6.95–6.90 (*m*, 2H), 3.81 (*s*, 3H). ^13^C NMR (151 MHz, CDCl_3_): δ 161.86, 156.52, 150.11, 148.07, 137.77, 131.16, 126.43, 122.44, 121.37, 114.38, 55.62. *m*/*z* (MS): [*M*]^+^ 228.00.

## 7. Refinement

Crystal data, data collection and structure refinement details are summarized in Table 2 ▸. All the hydrogen atoms were located in difference-Fourier maps and reﬁned using an isotropic approximation.

## Supplementary Material

Crystal structure: contains datablock(s) I. DOI: 10.1107/S2056989024010843/dx2062sup1.cif

Structure factors: contains datablock(s) I. DOI: 10.1107/S2056989024010843/dx2062Isup2.hkl

Supporting information file. DOI: 10.1107/S2056989024010843/dx2062Isup3.cml

CCDC reference: 2401430

Additional supporting information:  crystallographic information; 3D view; checkCIF report

## Figures and Tables

**Table 1:** Hydrogen-bond geometry (Å, °) *Cg*1 is the centroid of the C7–C12 ring.

*D*—H⋯*A*	*D*—H	H⋯*A*	*D*⋯*A*	*D*—H⋯*A*
N2—H2⋯N1	0.86	2.18	2.635 (4)	113
C1—H1⋯O1^i^	0.93	2.58	3.500 (4)	173
C3—H3⋯N1^ii^	0.93	2.74	3.402 (4)	129
C8—H8⋯O1	0.93	2.37	2.947 (4)	120
C11—H11⋯O2^iii^	0.93	2.63	3.558 (4)	173
C13—H13*C*⋯O1^iv^	0.96	2.51	3.468 (4)	173
C13—H13*B*⋯*Cg*1^v^	0.96	2.71	3.500 (4)	140

Symmetry codes: (i) 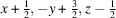; (ii) 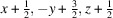; (iii) 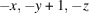; (iv) 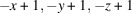; (v) 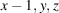.

**Table 2:** Experimental details

Crystal data	
Chemical formula	C_13_H_12_N_2_O_2_
*M*_r_	228.25
Crystal system, space group	Monoclinic, *P*2_1_/*n*
Temperature (K)	293
*a*, *b*, *c* (Å)	5.0082 (9), 20.728 (4), 11.1549 (14)
β (°)	96.998 (15)
*V* (Å^3^)	1149.3 (3)
*Z*	4
Radiation type	Cu *K*α
μ (mm^−1^)	0.74
Crystal size (mm)	0.10 × 0.08 × 0.07

Data collection	
Diffractometer	Xcalibur, Ruby
Absorption correction	Multi-scan (*CrysAlis PRO*; Rigaku OD, 2021 ▸)
*T*_min_, *T*_max_	0.669, 1.000
No. of measured, independent and observed [*I* > 2σ(*I*)] reflections	7362, 2166, 975
*R*_int_	0.096
(sin θ/λ)_max_ (Å^−1^)	0.609

Refinement	
*R*[*F*^2^ > 2σ(*F*^2^)], *wR*(*F*^2^), *S*	0.061, 0.139, 1.00
No. of reflections	2166
No. of parameters	156
H-atom treatment	H-atom parameters constrained
Δρ_max_, Δρ_min_ (e Å^−3^)	0.15, −0.16

Computer programs: *CrysAlis PRO* (Rigaku OD, 2021 ▸), *SHELXT* (Sheldrick, 2015*a* ▸), *SHELXL2016/6* (Sheldrick, 2015*b* ▸) and *OLEX2* (Dolomanov *et al.*, 2009 ▸).

## supplementary crystallographic information

### N-(4-Methoxyphenyl)pyridine-2-carboxamide Crystal data

**Table:**

C_13_H_12_N_2_O_2_	*F*(000) = 480
*M**_r_* = 228.25	*D*_x_ = 1.319 Mg m^−^^3^
Monoclinic, *P*2_1_/*n*	Cu *K*α radiation, λ = 1.54184 Å
*a* = 5.0082 (9) Å	Cell parameters from 638 reflections
*b* = 20.728 (4) Å	θ = 4.3–50.5°
*c* = 11.1549 (14) Å	µ = 0.74 mm^−^^1^
β = 96.998 (15)°	*T* = 293 K
*V* = 1149.3 (3) Å^3^	Block, colourless
*Z* = 4	0.10 × 0.08 × 0.07 mm

### N-(4-Methoxyphenyl)pyridine-2-carboxamide Data collection

**Table:**

Xcalibur, Ruby diffractometer	2166 independent reflections
Radiation source: fine-focus sealed X-ray tube, Enhance (Cu) X-ray Source	975 reflections with *I* > 2σ(*I*)
Graphite monochromator	*R*_int_ = 0.096
Detector resolution: 10.2576 pixels mm^-1^	θ_max_ = 70.0°, θ_min_ = 4.5°
ω scans	*h* = −6→6
Absorption correction: multi-scan (CrysAlisPro; Rigaku OD, 2021)	*k* = −23→25
*T*_min_ = 0.669, *T*_max_ = 1.000	*l* = −13→9
7362 measured reflections	

### N-(4-Methoxyphenyl)pyridine-2-carboxamide Refinement

**Table:**

Refinement on *F*^2^	Hydrogen site location: inferred from neighbouring sites
Least-squares matrix: full	H-atom parameters constrained
*R*[*F*^2^ > 2σ(*F*^2^)] = 0.061	*w* = 1/[σ^2^(*F*_o_^2^) + (0.0373*P*)^2^] where *P* = (*F*_o_^2^ + 2*F*_c_^2^)/3
*wR*(*F*^2^) = 0.139	(Δ/σ)_max_ < 0.001
*S* = 1.00	Δρ_max_ = 0.15 e Å^−^^3^
2166 reflections	Δρ_min_ = −0.16 e Å^−^^3^
156 parameters	Extinction correction: *SHELXL2016/6* (Sheldrick, 2015b), Fc^*^=kFc[1+0.001xFc^2^λ^3^/sin(2θ)]^-1/4^
0 restraints	Extinction coefficient: 0.0017 (4)
Primary atom site location: dual	

### N-(4-Methoxyphenyl)pyridine-2-carboxamide Special details

**Table:**

Geometry. All esds (except the esd in the dihedral angle between two l.s. planes) are estimated using the full covariance matrix. The cell esds are taken into account individually in the estimation of esds in distances, angles and torsion angles; correlations between esds in cell parameters are only used when they are defined by crystal symmetry. An approximate (isotropic) treatment of cell esds is used for estimating esds involving l.s. planes.

### N-(4-Methoxyphenyl)pyridine-2-carboxamide Fractional atomic coordinates and isotropic or equivalent isotropic displacement parameters (Å^2^)

**Table:**

	*x*	*y*	*z*	*U*_iso_*/*U*_eq_	
O1	0.7804 (5)	0.64248 (12)	0.5355 (2)	0.0708 (8)	
O2	−0.0904 (5)	0.46914 (13)	0.1774 (2)	0.0732 (8)	
N2	0.6732 (5)	0.65548 (14)	0.3324 (2)	0.0598 (8)	
H2	0.709512	0.679337	0.273443	0.072*	
N1	1.0276 (6)	0.74910 (15)	0.3271 (2)	0.0601 (8)	
C10	0.0926 (7)	0.51451 (18)	0.2243 (3)	0.0586 (10)	
C6	0.8116 (7)	0.66959 (18)	0.4398 (3)	0.0578 (10)	
C7	0.4767 (6)	0.60741 (18)	0.3013 (3)	0.0551 (10)	
C5	1.0171 (7)	0.72159 (18)	0.4350 (3)	0.0527 (9)	
C4	1.1852 (7)	0.73839 (18)	0.5373 (3)	0.0595 (10)	
H4	1.171623	0.718024	0.610576	0.071*	
C9	0.1619 (7)	0.52633 (19)	0.3453 (3)	0.0648 (11)	
H9	0.079459	0.503135	0.401950	0.078*	
C1	1.2108 (7)	0.79571 (19)	0.3221 (3)	0.0677 (11)	
H1	1.220637	0.815677	0.248061	0.081*	
C3	1.3734 (7)	0.78589 (19)	0.5287 (3)	0.0661 (11)	
H3	1.491329	0.797526	0.596193	0.079*	
C8	0.3529 (7)	0.5724 (2)	0.3835 (3)	0.0656 (11)	
H8	0.398288	0.579727	0.465682	0.079*	
C12	0.4052 (7)	0.59542 (19)	0.1794 (3)	0.0690 (11)	
H12	0.486200	0.618804	0.122515	0.083*	
C2	1.3864 (7)	0.81602 (19)	0.4202 (3)	0.0688 (11)	
H2A	1.509095	0.849017	0.412757	0.083*	
C11	0.2162 (7)	0.5494 (2)	0.1416 (3)	0.0730 (12)	
H11	0.171226	0.541804	0.059426	0.088*	
C13	−0.2421 (7)	0.43698 (19)	0.2588 (3)	0.0778 (12)	
H13A	−0.372539	0.409360	0.214373	0.117*	
H13B	−0.332549	0.468255	0.302912	0.117*	
H13C	−0.123774	0.411569	0.314264	0.117*	

### N-(4-Methoxyphenyl)pyridine-2-carboxamide Atomic displacement parameters (Å^2^)

**Table:**

	*U*^11^	*U*^22^	*U*^33^	*U*^12^	*U*^13^	*U*^23^
O1	0.0822 (19)	0.079 (2)	0.0492 (14)	−0.0123 (15)	0.0019 (13)	0.0042 (13)
O2	0.0762 (18)	0.077 (2)	0.0654 (16)	−0.0158 (16)	0.0045 (14)	−0.0085 (14)
N2	0.071 (2)	0.066 (2)	0.0402 (15)	−0.0061 (17)	−0.0033 (15)	0.0030 (14)
N1	0.068 (2)	0.065 (2)	0.0453 (17)	0.0046 (18)	0.0012 (14)	0.0028 (15)
C10	0.055 (2)	0.063 (3)	0.056 (2)	−0.002 (2)	0.0000 (18)	−0.0060 (19)
C6	0.064 (2)	0.060 (3)	0.049 (2)	0.007 (2)	0.0013 (18)	−0.0038 (18)
C7	0.056 (2)	0.057 (3)	0.050 (2)	0.004 (2)	−0.0028 (18)	−0.0055 (18)
C5	0.057 (2)	0.055 (3)	0.0440 (19)	0.0066 (19)	0.0002 (17)	−0.0019 (17)
C4	0.064 (2)	0.069 (3)	0.0440 (19)	0.000 (2)	0.0012 (18)	0.0016 (18)
C9	0.064 (2)	0.077 (3)	0.055 (2)	−0.008 (2)	0.0143 (19)	−0.003 (2)
C1	0.075 (3)	0.072 (3)	0.057 (2)	0.006 (2)	0.012 (2)	0.011 (2)
C3	0.066 (2)	0.078 (3)	0.051 (2)	−0.008 (2)	−0.0039 (19)	−0.006 (2)
C8	0.068 (3)	0.083 (3)	0.046 (2)	−0.002 (2)	0.0065 (19)	−0.007 (2)
C12	0.082 (3)	0.072 (3)	0.051 (2)	−0.014 (2)	−0.004 (2)	0.0076 (19)
C2	0.074 (3)	0.071 (3)	0.062 (2)	−0.012 (2)	0.010 (2)	−0.005 (2)
C11	0.085 (3)	0.083 (3)	0.047 (2)	−0.014 (2)	−0.008 (2)	0.003 (2)
C13	0.064 (3)	0.078 (3)	0.092 (3)	−0.009 (2)	0.013 (2)	−0.001 (2)

### N-(4-Methoxyphenyl)pyridine-2-carboxamide Geometric parameters (Å, º)

**Table:**

O1—C6	1.233 (4)	C4—C3	1.374 (5)
O2—C10	1.371 (4)	C9—H9	0.9300
O2—C13	1.419 (4)	C9—C8	1.381 (5)
N2—H2	0.8600	C1—H1	0.9300
N2—C6	1.341 (4)	C1—C2	1.384 (4)
N2—C7	1.413 (4)	C3—H3	0.9300
N1—C5	1.339 (4)	C3—C2	1.371 (4)
N1—C1	1.338 (4)	C8—H8	0.9300
C10—C9	1.374 (4)	C12—H12	0.9300
C10—C11	1.378 (5)	C12—C11	1.373 (5)
C6—C5	1.496 (5)	C2—H2A	0.9300
C7—C8	1.376 (5)	C11—H11	0.9300
C7—C12	1.386 (4)	C13—H13A	0.9600
C5—C4	1.378 (4)	C13—H13B	0.9600
C4—H4	0.9300	C13—H13C	0.9600
			
C10—O2—C13	117.6 (3)	N1—C1—C2	124.0 (3)
C6—N2—H2	115.0	C2—C1—H1	118.0
C6—N2—C7	129.9 (3)	C4—C3—H3	120.2
C7—N2—H2	115.0	C2—C3—C4	119.6 (3)
C1—N1—C5	116.6 (3)	C2—C3—H3	120.2
O2—C10—C9	125.0 (3)	C7—C8—C9	120.7 (3)
O2—C10—C11	116.0 (3)	C7—C8—H8	119.6
C9—C10—C11	118.9 (3)	C9—C8—H8	119.6
O1—C6—N2	124.6 (4)	C7—C12—H12	119.6
O1—C6—C5	121.3 (3)	C11—C12—C7	120.8 (4)
N2—C6—C5	114.1 (3)	C11—C12—H12	119.6
C8—C7—N2	124.4 (3)	C1—C2—H2A	121.1
C8—C7—C12	118.4 (3)	C3—C2—C1	117.8 (4)
C12—C7—N2	117.2 (3)	C3—C2—H2A	121.1
N1—C5—C6	116.2 (3)	C10—C11—H11	119.7
N1—C5—C4	123.4 (3)	C12—C11—C10	120.5 (3)
C4—C5—C6	120.4 (3)	C12—C11—H11	119.7
C5—C4—H4	120.7	O2—C13—H13A	109.5
C3—C4—C5	118.6 (3)	O2—C13—H13B	109.5
C3—C4—H4	120.7	O2—C13—H13C	109.5
C10—C9—H9	119.7	H13A—C13—H13B	109.5
C10—C9—C8	120.6 (3)	H13A—C13—H13C	109.5
C8—C9—H9	119.7	H13B—C13—H13C	109.5
N1—C1—H1	118.0		
			
O1—C6—C5—N1	−177.9 (3)	C7—N2—C6—O1	−2.8 (6)
O1—C6—C5—C4	2.9 (5)	C7—N2—C6—C5	176.1 (3)
O2—C10—C9—C8	178.7 (3)	C7—C12—C11—C10	0.4 (6)
O2—C10—C11—C12	−179.1 (3)	C5—N1—C1—C2	0.4 (5)
N2—C6—C5—N1	3.1 (4)	C5—C4—C3—C2	1.2 (5)
N2—C6—C5—C4	−176.1 (3)	C4—C3—C2—C1	−1.6 (6)
N2—C7—C8—C9	179.8 (3)	C9—C10—C11—C12	−0.1 (6)
N2—C7—C12—C11	179.9 (3)	C1—N1—C5—C6	179.9 (3)
N1—C5—C4—C3	0.1 (5)	C1—N1—C5—C4	−0.9 (5)
N1—C1—C2—C3	0.8 (6)	C8—C7—C12—C11	−0.3 (6)
C10—C9—C8—C7	0.2 (6)	C12—C7—C8—C9	0.0 (6)
C6—N2—C7—C8	12.7 (6)	C11—C10—C9—C8	−0.2 (6)
C6—N2—C7—C12	−167.5 (3)	C13—O2—C10—C9	7.5 (5)
C6—C5—C4—C3	179.2 (3)	C13—O2—C10—C11	−173.6 (3)

### N-(4-Methoxyphenyl)pyridine-2-carboxamide Hydrogen-bond geometry (Å, º)

*Cg*1 is the centroid of the C7–C12 ring.

**Table:**

*D*—H···*A*	*D*—H	H···*A*	*D*···*A*	*D*—H···*A*
N2—H2···N1	0.86	2.18	2.635 (4)	113
C1—H1···O1^i^	0.93	2.58	3.500 (4)	173
C3—H3···N1^ii^	0.93	2.74	3.402 (4)	129
C8—H8···O1	0.93	2.37	2.947 (4)	120
C11—H11···O2^iii^	0.93	2.63	3.558 (4)	173
C13—H13*C*···O1^iv^	0.96	2.51	3.468 (4)	173
C13—H13*B*···*Cg*1^v^	0.96	2.71	3.500 (4)	140

Symmetry codes: (i) *x*+1/2, −*y*+3/2, *z*−1/2; (ii) *x*+1/2, −*y*+3/2, *z*+1/2; (iii) −*x*, −*y*+1, −*z*; (iv) −*x*+1, −*y*+1, −*z*+1; (v) *x*−1, *y*, *z*.
